# Objective Prediction of Next-Day’s Affect Using Multimodal Physiological and Behavioral Data: Algorithm Development and Validation Study

**DOI:** 10.2196/39425

**Published:** 2023-03-15

**Authors:** Salar Jafarlou, Jocelyn Lai, Iman Azimi, Zahra Mousavi, Sina Labbaf, Ramesh C Jain, Nikil Dutt, Jessica L Borelli, Amir Rahmani

**Affiliations:** 1 Donald Bren School of Information and Computer Sciences University of California, Irvine Irvine, CA United States; 2 Department of Psychological Science University of California, Irvine Irvine, CA United States; 3 Institute for Future Health University of California, Irvine Irvine, CA United States; 4 Department of Cognitive Science University of California, Irvine Irvine, CA United States; 5 School of Nursing University of California, Irvine Irvine, CA United States

**Keywords:** wearable devices, mental health, affective computing

## Abstract

**Background:**

Affective states are important aspects of healthy functioning; as such, monitoring and understanding affect is necessary for the assessment and treatment of mood-based disorders. Recent advancements in wearable technologies have increased the use of such tools in detecting and accurately estimating mental states (eg, affect, mood, and stress), offering comprehensive and continuous monitoring of individuals over time.

**Objective:**

Previous attempts to model an individual’s mental state relied on subjective measurements or the inclusion of only a few objective monitoring modalities (eg, smartphones). This study aims to investigate the capacity of monitoring affect using fully objective measurements. We conducted a comparatively long-term (12-month) study with a holistic sampling of participants’ moods, including 20 affective states.

**Methods:**

Longitudinal physiological data (eg, sleep and heart rate), as well as daily assessments of affect, were collected using 3 modalities (ie, smartphone, watch, and ring) from 20 college students over a year. We examined the difference between the distributions of data collected from each modality along with the differences between their rates of missingness. Out of the 20 participants, 7 provided us with 200 or more days’ worth of data, and we used this for our predictive modeling setup. Distributions of positive affect (PA) and negative affect (NA) among the 7 selected participants were observed. For predictive modeling, we assessed the performance of different machine learning models, including random forests (RFs), support vector machines (SVMs), multilayer perceptron (MLP), and K-nearest neighbor (KNN). We also investigated the capability of each modality in predicting mood and the most important features of PA and NA RF models.

**Results:**

RF was the best-performing model in our analysis and performed mood and stress (nervousness) prediction with ~81% and ~72% accuracy, respectively. PA models resulted in better performance compared to NA. The order of the most important modalities in predicting PA and NA was the smart ring, phone, and watch, respectively. SHAP (Shapley Additive Explanations) analysis showed that sleep and activity-related features were the most impactful in predicting PA and NA.

**Conclusions:**

Generic machine learning–based affect prediction models, trained with population data, outperform existing methods, which use the individual’s historical information. Our findings indicated that our mood prediction method outperformed the existing methods. Additionally, we found that sleep and activity level were the most important features for predicting next-day PA and NA, respectively.

## Introduction

National estimates of mental health suggest that, as of 2019, 1 in 5 adults in the United States experiences mental disorders, with young adults at greater risk than their older counterparts [1]. An important facet of mental health is the affective, subjective, and physiological experiences of emotion that differ by valence (positive and negative states) [2,3]. Affective disturbances and dysregulation, more specifically, overly heightened or dampened affect, prolonged NA, or instability in experienced affect, are core facets of many types of psychopathology. Monitoring and increased understanding of one’s affect may contribute to the regulation of affect and, as such, are key components of many forms of intervention or approaches (eg, therapy or medication administration) to manage affective disturbances [4,5]. Given that monitoring and understanding affect are crucial for the treatment and management of mood-based disorders, it may be helpful to also consider the degree to which affective experiences serve as information regarding future mental well-being. Therefore, the prediction of future affect can allow preventative approaches for mitigating affective disturbances and dysregulation.

There are several reasons why it might be important to help individuals in predicting their affect. Along with emotion and mood, affect involves complex states with several response systems like behavioral expressions, physiological and neural reactivity, and subjectively felt experiences. Second, experiences of affect involve the appraisal or interpretation of one’s thoughts and the external environment (eg, running late to work and feeling frantic) that then elicits affective responses; once an emotion is elicited, there are also response tendencies, or ways in which people may respond to the affect or emotions felt (eg, speeding to get to work) [6]. Altogether, how affect may be elicited and experienced is quite complex, depending on the context, and it involves multiple response systems within an individual. Little research has examined individual differences in a person’s awareness of their own emotions. Identifying and having increased granularity for describing one’s own emotions are relevant factors in psychological well-being [7]. Despite these important associations, it is unlikely that individuals are constantly monitoring their affect, emotion, or mood throughout their day. Mood-tracking studies and other ecological momentary assessments (EMAs) suggest that, while helpful, individuals may find it a hassle to constantly pay attention to their emotions [8]. Using noninvasive wearables and other tools to capture experiences may help individuals in predicting when certain affective states may occur as well as help them regulate (eg, actively select or avoid situations that would not be adaptive for their mood).

Traditional therapeutic approaches to support emotion management rely on the patient’s self-report or monitoring through a diary. Patients may require assistance in interpreting their affect, emotions, or moods from a therapist or provider (eg, in identifying their feelings or the causes of their feelings). Existing tools, such as commercially available mood-tracking phone apps, and proposed digital tools have been developed to subjectively monitor emotion and mood [8-11]. With respect to studies examining emotion with greater ecological validity, studies have been conducted to collect EMAs, leveraging internet-based questionnaires. For example, Danowitz and Beddoes [12] conducted a semester-long study, collecting data through daily surveys and sampling the mental state of engineering students across 5 institutions in the United States. The surveys were designed to include overall mental health disorders (eg, depressive, anxiety, and eating disorders) and their symptoms in the targeted population. These solutions can enable a therapist to track the user’s mental health condition remotely or help with emotion management during sessions [13]. However, the proposed solutions were merely limited to questionnaires.

More recently, wearable devices and artificial intelligence-based methods have been used to perform mood analysis and prediction, resulting in a better understanding of an individual’s mental health state [14]. There have been several attempts to explore the feasibility of modeling people’s emotions, moods, and stress levels in laboratories or other controlled settings. A limited number of studies addressed mood prediction using a continual and daily approach [10,15,16]. For example, Wang et al [17] assessed sleep, activity, mood, and other factors affecting academic performance in 48 students throughout a 10-week time period in a college. They leveraged smartphone sensors (including the accelerometer [ACC], microphone, and light sensor) to perform context- and behavior-enabled mental health assessments. However, the study lacked an accurate assessment of individuals’ biological states. Another holistic study (ie, SNAPSHOT) [18] was introduced to monitor participants’ daily lives for 1 month using 2 wristbands, a smartphone, and self-report questionnaires. Sano [18] showed that indicators of an individual’s well-being (eg, mood, stress, and health) could be modeled using a set of subjective and objective behavioral and biological features. Using the collected data, Taylor et al [19] obtained 65.8% and 67.9% accuracy in predicting mood and stress for the next day, respectively.

The previously mentioned studies have presented mental health assessment and modeling approaches. However, they have mostly relied on users’ subjective evaluation inputs for affect and mood modeling. Unfortunately, such subjective data assessments require a user’s dedicated attention, resulting in an increased burden on users and unsatisfactory experiences. In addition, subjective assessments are more prone to higher rates of missing and inaccurate data, as the users might forget to report when prompted and rely on the recall of past experiences. Subjective data collection methods are also limited to certain times throughout the monitoring phase. Therefore, subjective assessments are unable to provide ubiquitous monitoring, resulting in the absence of information between assessments. Another issue with the existing work is that it was limited to short-term or midterm data collection (ie, a few weeks to a few months). Such approaches have not captured long-term changes or responses to certain stressors and events (eg, the COVID-19 pandemic) throughout the monitoring period. Another shortcoming of the current wearable-based mental health assessment study is that it predicts only a few psychological and well-being factors. Further, they are mostly limited to a few target labels, such as stress, depression, and personality disorder [20-22], although having an accurate evaluation of one’s psychological well-being requires a comprehensive evaluation of their mental state.

The advances in information and communication technologies, for example, the Internet of Things (IoT), wearable IoT, and machine learning, have opened new gateways to track an individual’s health condition uninterruptedly [23]. However, deploying these technologies in mental health assessment is still in its early stages. IoT-based solutions have not been fully leveraged in existing studies, both for ubiquitous monitoring and for modeling the dynamics of an individual’s mental state over time. We believe that such emerging technologies provide an unprecedented opportunity to track mental health in everyday settings. To this end, we aim to tailor them to address the limitations of current affect monitoring and assessment by enabling holistic and long-term data collection and analysis.

In this paper, we introduce an IoT-based platform (named ZOTCARE) to monitor the physiological and behavioral states of individuals along with a subjective evaluation of their mental health and affective state. We conducted a 12-month study on 20 college students, during pre- and post–COVID-19 lockdown. The participants were recruited to continuously wear smart watches and rings and use an activity detection mobile app to monitor their physiological and behavioral states. In addition, they were prompted daily with a smartphone-based EMA questionnaire to report their perceived affective states. We developed an affect prediction method, enabled by multimodal physiological and behavioral parameters. To this end, we used and evaluated a set of machine learning models.

## Methods

In this section, we discuss different aspects of our longitudinal study for affect prediction, including data collection setup, features and labels, and the affect prediction modeling scheme.

### Data Collection Setup

To develop our fully objective models, we collected data from 20 college students (n=13, 65% female participants; mean_age_ 19.80, SD_age_ 1.0 years) as part of a larger study aimed at assessing personalized approaches to understanding mental health and well-being among emerging adults [1]. Participants were eligible if they were (1) full-time college students between the ages of 18 and 22 years, (2) unmarried, (3) fluent in English, and (4) Android phone device users. This extensive longitudinal study was conducted over a 12-month period among students at a large West Coast university. The data were collected throughout 2020 during the COVID-19 pandemic. The college students were asked to wear the Samsung Galaxy Active 2 watch along with the Oura ring [17] second generation, which captured physiological, sleep, and physical activity parameters. These noninvasive commercial smart devices were equipped with sensors, including photoplethysmography (PPG) optical sensors, ACCs, gyroscopes, and body temperature sensors. The students downloaded a smartphone app that detected activities and life logs (eg, staying still, walking, and in-vehicle), movement, and location. During this monitoring period, participants also completed daily surveys using a separate smartphone app that prompted them to report their affect at the end of the day based on a list of 20 discrete emotion words. The daily self-reported affect was used to evaluate our predictive models.

**Figure 1:**
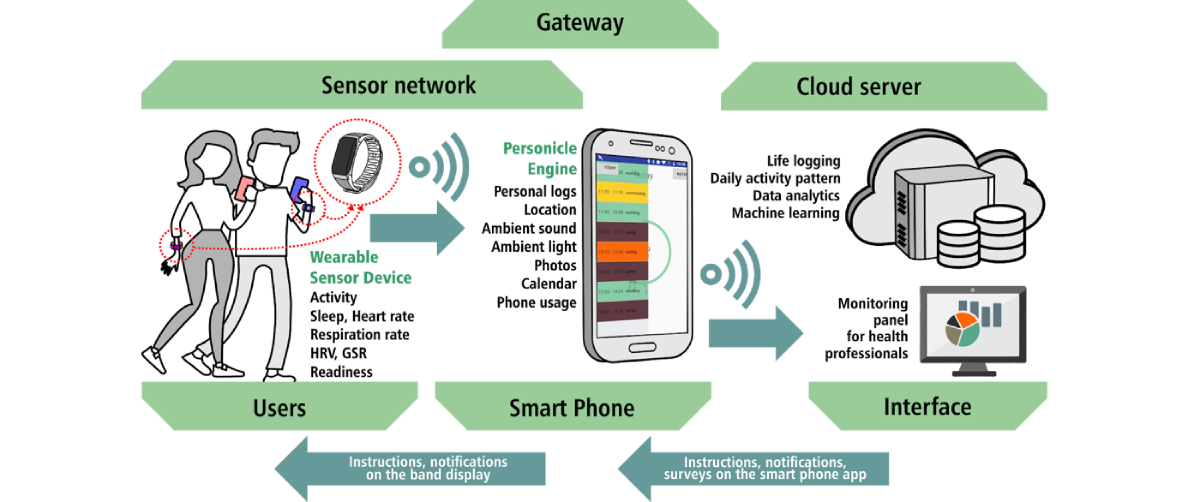
Data collection setup; wearable devices collect targeted information through apps we developed and send the data to our cloud server by proxying the smartphone’s internet connection. Smartphones are also used to collect daily affect assessments from participants. GSR: galvanic skin response; HRV: heart rate variability.

The collected data were transferred and stored in real time using a number of services offered by ZotCare [13]. ZotCare is a dynamic and flexible multilayer (sensor-smartphone-cloud) platform, built at the University of California, Irvine Institute for Future Health that provides a variety of services to run holistic human study trials. Different layers and data flow of the ZotCare are shown in Figure 1. Subjective mood assessment data were collected using ZotCare’s mobile app.

### Ethics Approval

This study was approved by the University of California, Irvine's Institutional Review Board (HS#2019-5153). Participants provided written consent prior to their participation in the study. Because this investigation was part of a larger study examining mental health trajectories over time, eligible participants were first screened for severe depression or suicidal ideation and consulted with a team member of the project who was a clinical psychologist. Participants were allowed to withdraw from the study, and the principal investigators could determine whether to discontinue the participant’s involvement in the study to ensure their health and safety at any time. For participating, the individuals were compensated up to US $260, depending on whether they completed all aspects of the study (eg, baseline assessment, longitudinal monitoring, several mid point and end point assessments).

### Modalities and Labels

We obtained 3 data modalities from the smart ring, watch, and phone. We outline the modalities as follows.

#### Smart Ring

This modality includes information about sleep (eg, length of awake, deep, and rapid eye movement [REM] sleep stages), physiology (eg, heart rate, heart rate variability), and the activity of the users (eg, daily movement and rest time) collected by Oura smart rings. In our study, the information extracted from this modality is chronically high-level (day-level). From the viewpoint of missing data, the convenience of wearing the ring and Oura’s built-in data management make this modality more continuous and reliable [24].

#### Smart Watch

This modality contains fine-grained physiological data of users. In contrast to smart rings, smart watches are capable of recording and storing raw signals with a higher frequency and resolution [24]. We recorded raw ACC, gyroscope, and PPG signals in 12-minute windows every 2 hours throughout the day. These high-resolution raw signals can be processed to extract valuable measures such as HRV, which is correlated with nervous system responses. However, this modality requires a higher power consumption device, thus requiring more frequent charging and, therefore, a higher likelihood of missing data.

#### Smartphone

We also monitored the users’ activity types and location changes as a separate modality using the Personicle Android monitoring app [16]. The Personicle app collected major physical (eg, in a vehicle, still, on a bicycle) and behavioral (eg, working, commuting, and relaxing) activity data throughout the day using the Android application programming interface. This modality relies mostly on the smartphone’s movement and location to detect these activities. It should be noted that most of the in-house activities might be missed due to the lockdown situation and the movement limitations of the participants.

Finally, using these 3 modalities, we collected 52 features**,** as shown in Textbox 1, each of which was captured daily or intensively (eg, a 5-minute sliding average for the ring) over the course of the day. The features captured intensively were weighted-averaged, with respect to the duration.

**List of collected features based on modality (device) and objectives. For further information about ring and watch features, please refer to their web-based documentation [14-16].**

**Ring**

- Sleep (mins)
  Awake
  Rapid eye movement
  Light
  Deep
  Total
- Activity
  Stay active
  Meet daily activity target
  Move every hour
  Training frequency
  Training volume
  Recovery time
  Daily movement
  Inactivity alerts
- Metabolic
  Average metabolic equivalent minutes (MET)
  MET inactive
  Minutes low activity
  MET low
  Minutes medium activity
  MET medium
  Minutes high activity
  MET high
- Calorie
  Calorie active
  Calorie total
  Target calories
  Target miles
- Heart
  Heart rate
  Heart rate std
  Heart rate variability std

**Watch**

- Distance
  Distance
  Run steps
  Remains
  Walk steps
- Environment
  Pressure
  Pressure min
  Pressure max

**Phone**

- Activity
  Main activity
  Key activity
  Location change

### Affect Labels (Daily Assessment)

For the daily assessments of affect, the participants were asked to rate, on a scale of 0-100 (0=“Very Slightly; 100=“Extremely”) how they felt on a series of 20 different discrete emotion items (eg, “inspired,” “enthusiastic,” “nervous,” and “upset”) over the course of the day. The selected items were adapted from the Positive and Negative Affect Schedule [18], a frequently used scale to assess emotions. Each emotion was examined separately but also as a composite. For example, PA was calculated as the average of 10 positive affective items (mean 45.27, SD 20.22; α=.85), and NA was calculated as the average of 10 negative affective items (mean 21.79, SD 12.28; α=.91).

### Predictive Modeling Scheme

**Figure 2:**
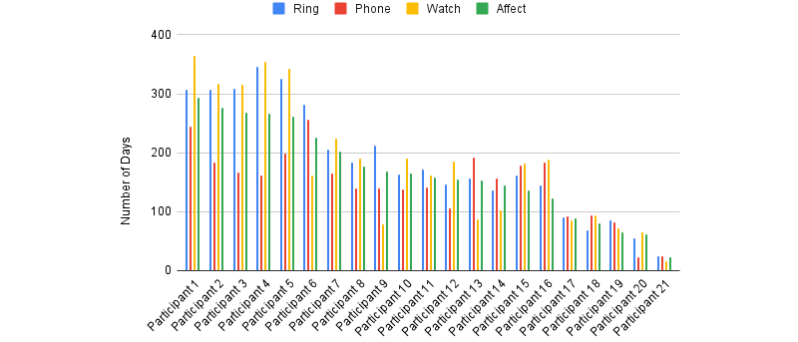
The number of days that participants had valid data collected from different devices and ecological momentary assessment (EMA) sorted by affect values.

We developed machine learning methods to classify different emotions. The binary classification label was defined as predicting if each emotion value was above or below the median of the whole distribution of all participants. Therefore, the obtained binary labels were balanced. The middle 20% of the values were removed from the whole distribution before the class labeling [25]. We used random forest (RF), support vector machine (SVM), multilayer perceptron (MLP), and K-nearest neighbor (KNN) models for the prediction. We selected the participants with more than 200 days of affect data available. This included 7 participants out of 20 (see Figure 2). In the preprocessing phase, detected activities by Personicle were converted to continuous values, each of which represents the weighted average in time during the day. Features were *z*-normalized, with respect to the mean and variance of the training set, and fed to the model. The hyper-parameters of each model were tuned with respect to the accuracy of the validation set (ie, a 10% held-out portion of the data set). We performed random 5-fold cross-validation on the remaining 90% of the data set, and the accuracies were averaged out. We selected PA as the closest label to compare our models with other related works [18], in which an aggregate single binary value is used to represent the emotional state (ie, sad or happy) [19]. The nervousness label in the questionnaire we referred to as stress, which has been used interchangeably in related works [19].

## Results

### Affect Feature and Label Analysis

In this section, we examined the characteristics of the features collected from different modalities and labels. We first indicated the distribution differences of the selected 8 features, that is, 4 physiological, 2 physical activity, and 2 behavioral parameters. We selected 7 out of 20 participants with more than 200 days of valid affect scores. We then showed the PA and NA label distributions of the 7 participants. Finally, the distributions of missing data, separated by their modalities and participants, are presented.

**Figure 3:**
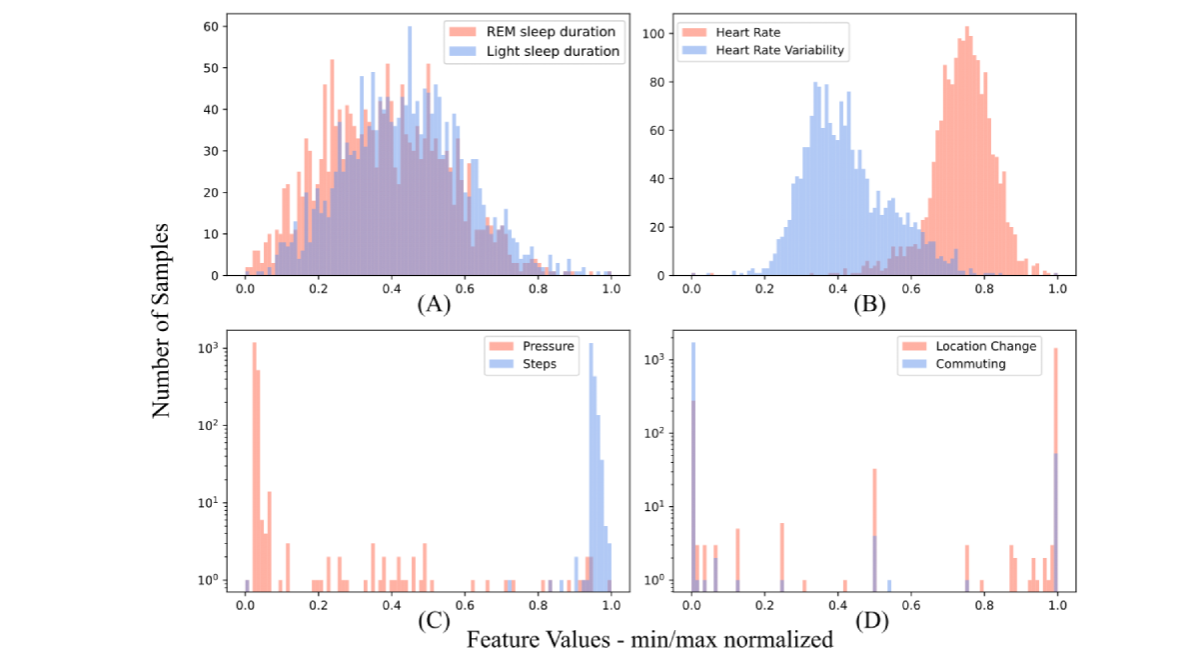
Histogram of selected features. (A) REM and light sleep durations; (B) heart rate and heart rate variability collected by ring; (C) atmospheric pressure (altitude) and step counts collected by smart watch; and (D) location change and commute events detected by the Personicle app on the phone. REM: rapid eye movement.

The distributions of features selected from different modalities are shown in Figure 3. We observed that features related to physiological states tended to have data samples within their entire value range compared to features related to physical activity and activity detection, which generally had a sparse distribution across their feature space. Figure 3A,B indicates the (min and max normalized) distributions of REM and light sleep, heart rate, and heart rate variability collected by the Oura ring. Figure 3C depicts atmospheric pressure and the number of steps detected by the smart watch. The distribution of the number of steps after min-max normalization is very concentrated (the y axis is logarithmic), showing the users had intensive physical activities on certain days. This could be an effect of the COVID-19 lockdown circumstances. Finally, the distributions of location changes and commuting features collected via the Personicle app are shown in Figure 3D. Reliance of this modality on a smartphone’s GPS and motion sensors made this modality largely affected by the pandemic lockdown situation, as the users tended to communicate and move less than usual.

**Figure 4:**
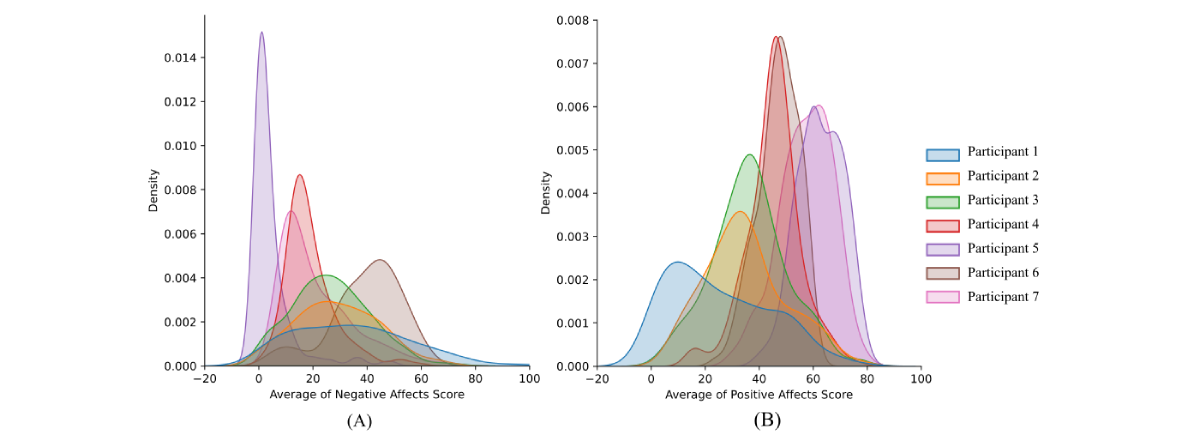
Smoothed distribution of (A) negative affect (NA) and (B) averaged positive affect (PA) of 7 selected participants. NAs tend to have a narrower distribution compared to PAs.

Figure 4 demonstrates the averaged affect distributions for participants with more than 200 days of valid affect values. In general, NA (Figure 3A) had a narrower distribution with higher peaks; this makes NA score values less discriminative compared to PA (Figure 3B). We define classification labels if each day’s affect is above or below the median of the distribution. For example, 2 days with similar NA scores could have small differences but get labeled differently because they are located on different sides of the median (refer to the previous section for the classification scheme).

Missing data is a major challenge in our 1-year human subject study performed in everyday settings as well as during the COVID-19 pandemic. In particular, the increased volume of missing data poses additional challenges for data modeling. Figure 4 shows the number of days of available data, divided by participants and modalities. We found that activities detected by smartphone data (Personicle) were the modality with the highest ratio of missing values. We speculate that when the participants spent most of their time at home (self-quarantining), the movement data were missing, as, for example, when they left their smartphones at their desks. The rings and watches generally had the fewest missing values, confirming the necessity of objective measurements through wearable devices. For affect prediction, we handled missing data using a single imputation method [26,27]. To this end, the missing value was interpolated using the data of the 2 preceding and succeeding days with valid data. No data imputation was applied to the targeted affect scores to avoid inaccuracy in the prediction.

### Affect Prediction

**Figure 5:**
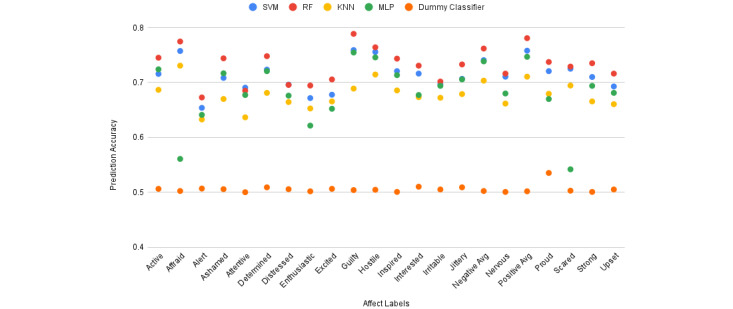
Accuracy of RF, SVM, KNN, and MLP models and a dummy classifier calculated in 5-fold cross-validation. KNN: K-nearest neighbor; MLP: multilayer perceptron; RF: random forest; SVM: support vector machine.

As previously mentioned, we used machine learning methods to perform daily affect prediction. In the following, we evaluated the performance of 4 machine learning models on each reported affect score. Figure 5 demonstrates the accuracy of the models and a dummy classifier using a 5-fold cross-validation method. The accuracy of SVM, MLP, and KNN is similar. However, RF outperformed the other models in terms of accuracy across all affect labels. “Guilty” and “Alert” had the most and least prediction accuracy, respectively, with 78.8% and 67.2%. We found that PA had higher predictive accuracy than NA (78.1% vs 76.1% accuracies). The best accuracy achieved for the nervousness label was 71.6%. In the rest of the paper, we select RF as the classifier to perform feature and label analysis.

**Figure 6:**
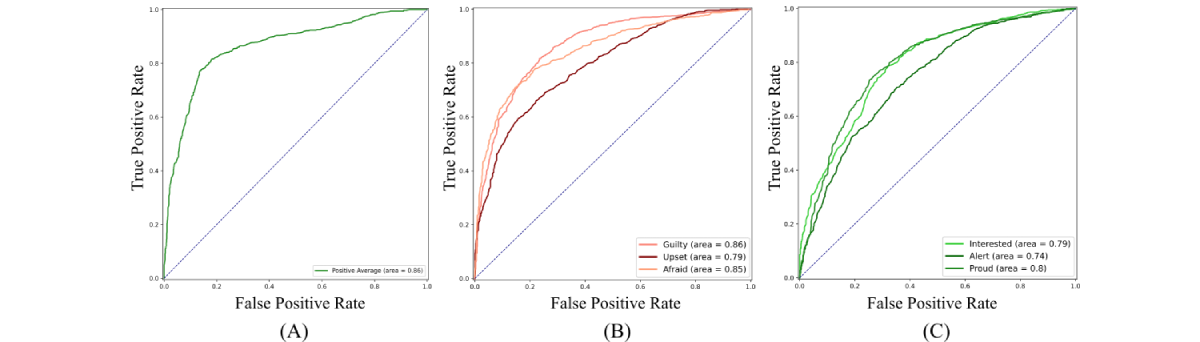
Receiver operating characteristic for compound positive affect (A), 3 negative affect (B), and 3 positive affect (C) with the largest, medium, and lowest area under the curves.

Figure 6 shows the receiver operating characteristic (ROC) and area under the curve (AUC) of RF classifier performances for different labels. Figure 6A indicates the ROC of the model predicting mood as a general indicator. Figures 6B and C demonstrate the ROC of affect prediction models with the highest, lowest, and median AUC within NAs and PAs, respectively. NA models had a higher ROC but, in general, lower accuracy (Table 1). The main reason could be the comparatively narrower distribution of NA (Figure 3A) that results in closer values getting different labels. This indicates that despite better probabilities being generated for NA, the chance of producing the wrong label is higher since the labeling thresholds are narrower.

One of the objectives of this study was to explore the capabilities of leveraging multimodal data for modeling affect. In general, multimodal machine learning could be used to either increase robustness (ie, by providing redundant information) or improve prediction performance (ie, by obtaining additional information from different aspects of the event). In this study, we focused on the latter purpose of multimodal machine learning. Therefore, assessment modalities were selected to monitor different aspects of an individual’s life, from physiological (ring and watch) to behavioral (phone) parameters. To test the power of multimodal assessment, we trained the model’s modalities separately and all together. Table 1 compares the accuracy of RF models trained with data from different modalities separately to ones trained with data from all devices for NA and PA. In single-modality setups, the smart ring, phone, and watch have, respectively, the highest capability to predict both PA and NA. We also show that leveraging all of the modalities improves accuracy by up to 21.8%.

**Figure 7:**
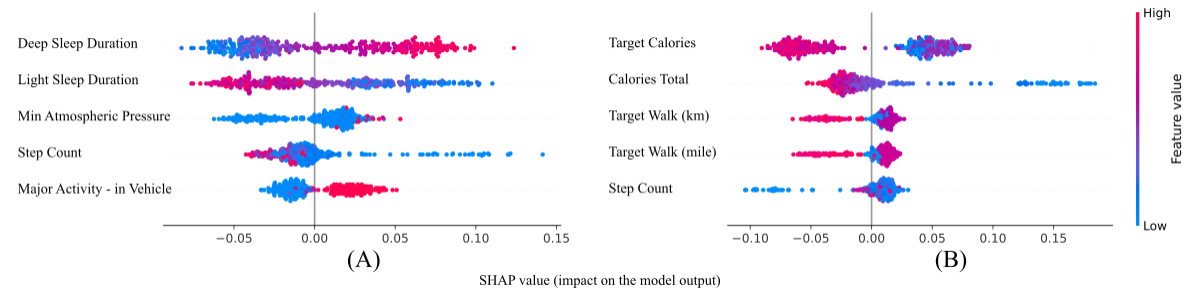
SHAP (Shapley Additive Explanations) values of the 5 most impactful features on positive affect (A) and negative affect (B) predictive models.

We explored the importance of each feature in the PA and NA predictive models. SHAP (Shapley Additive Explanations) [28] is a game theory-based method proposed to explain the effects of input features on machine learning model outputs. Figure 7 represents the SHAP values of the top 5 most impactful features on PA and NA RF models in the test set. We can see that high deep sleep and low light sleep length both have a clear impact on PA. We can also observe that higher “in vehicle” main activity has a distinctive effect on the PA outcome. Lower target and total calories have the most impact on the NA label. We can also see that higher target walking distance values distinctively affect NA prediction in a negative way. Step count appears among the top 5 most important features on both PA and NA.

**Table 1:** Accuracy (%) of trained random forest models on modalities separately and all together for positive affect and negative affect.

	All Modalities	Ring	Watch	Phone
Positive average	76.82	72.78	63.07	65.22
Negative average	74.47	71.49	61.55	62.13

## Discussion

### Principal Findings

The purpose of this study was to investigate the viability of predicting an individual’s next-day affect solely through objective measurements, without the need for user intervention to collect feedback. We used multimodal data collection methods for affect prediction tasks and found that the smart ring is the most reliable modality with the least amount of missing data. This modality also yielded the best accuracy in PA and NA prediction in single-modality experiments. The smartphone and watch were, respectively, the most predictive modalities in these affect.

Results showed that we can predict next-day PA with an accuracy of ~78% using generic machine learning algorithms. These models outperformed their alternatives in similar studies even without an individual’s historical information for personalization or temporal context. The results reveal that features extracted from each modality tend to have different distributions. The outcomes also confirm that leveraging multiple modalities together increases the affect modeling capability. Sleep and daily movement features collected by the smart ring are among the most important predictors of PA and NA, which suggests further investigation into the importance of sleep and movement on affect. Using objective approaches such as this can be expanded to do predictive modeling of mental health symptoms (eg depression, loneliness).

### Comparison With Prior Work

The SNAPSHOT data set is one of the similar works in the literature to our study [18]. Taylor et al [19] investigated mood, stress, and health prediction using a set of features in this data set. In that work, the authors leverage daily survey information collected about participants’ activity, sleep, and interactions. With a modeling scheme similar to our work, they achieved 65.8% and 67.9% of accuracy for mood and stress prediction models. We reported accuracy rates of 78.1% and 71.6% for PA and nervousness, respectively, which could be considered equivalent labels for their work. Multiple parameters in a real-world study make a fair comparison between 2 studies a difficult task. Our rich and continuous data collection method (specifically in sleep using a smart ring) was shown to be a strong candidate for subjective alternatives.

Spathis et al [29] reported similar daily mood classification modeling performance on the Emotion Sense [30] data set. The data set that Spathis and colleagues [29] used contained daily reports of participants’ moods along with information collected from their smartphones. They also collected information aimed to cover the environment, activity, and sociability factors of the participants’ daily lives. From our study, there was an 8.1% improvement in the AUC of the ROC in our experiments (Figure 6), compared to the best values reported by [29] for mood prediction.

### Limitations and Future Work

Our data collection was limited to Android smartphones. This reduces the external validity of the study because many college students use iOS. In future work, we would increase the generalizability of the work to be able to expand beyond Android users, though it would introduce new technological challenges. Another limitation was that, during the study, COVID-19 lockdown circumstances imposed some challenges for data collection due to limited contact with participants and disruptions to their normal movement and commute patterns. These challenges may have imposed a greater rate of missing data.

In our future work, we plan to investigate methods that incorporate patient-specific information into modeling. We are also planning to investigate deep-learning methods for automatic feature extraction from raw PPG and ACC data in the context of affect prediction. Our findings suggest that objective assessment in real-time can provide accurate information about people’s experiences. Future studies can include exploring the feasibility of interventions triggered by the outcomes of the predictive models to regulate and manage their affect. Future research may benefit from designing and refining interventions among emerging adults using smartphones. For instance, monitoring and prediction of affect could be used to identify when to use interventions in the hope of personalizing interventions targeting affect and other aspects of mental health.

### Conclusions

Tracking an individual’s affective state has shown to be a rich source of information vital to their mental well-being. Human psychology is intrinsically complex, making it difficult to monitor these states, especially in a continuous and uninterrupted way. Recent advancements in smart wearable devices, accompanied by novel machine learning methods, offer opportunities to predictably model affective states. This study investigated the viability of modeling affective states using wearable devices that objectively monitor certain parameters of users’ physiological and behavioral states. We conducted a 12-month study on 20 college students, collecting 20 of their daily affect. We monitored physiology and activities using smart wearable devices. We then investigated the characteristics of the collected data. We developed machine learning models to predict next-day affective states using objective measurements and investigated the most impactful factors in these predictions. We demonstrated the capabilities of multimodal and continuous data collection methods. Our generic personal models gained ~78% accuracy in PA prediction without requiring personalization techniques. Sleep and physical activity were shown to be among the most impactful parameters in determining an individual’s PA and NA.

## Abbreviations

ACC: accelerometer
AUC: area under the curve
EMA: ecological momentary assessment
IoT: Internet of Things
KNN: K-nearest neighbor
MLP: multilayer perceptron
NA: negative affect
PA: positive affect
PPG: photoplethysmography
REM: rapid eye movement
RF: random forest
ROC: receiver operating characteristic
SHAP: Shapley Additive Explanations
SVM: support vector machine
